# Improving Female Participation in Professional Engineering Geology to Bring New Perspectives to Ethics in the Geosciences

**DOI:** 10.3390/ijerph110909429

**Published:** 2014-09-11

**Authors:** Dolores Pereira

**Affiliations:** Departamento de Geología, Facultad de Ciencias, University of Salamanca, Plaza de la Merced s/n, 37008 Salamanca, Spain; E-Mail: mdp@usal.es; Tel.: +34-9-2329-4498; Fax: +34-9-2329-4514

**Keywords:** engineering geology, female participation, role models/mentoring, engineering studies, final project

## Abstract

Many papers have been published related to the retention and advancement of women in sciences. Engineering geology is one of the professional areas where women have not yet broken the gender barrier. The research issues of this paper are focused on why female students “leak out” at the end of engineering geology studies, and what can be done to encourage them to complete their degrees with an engineering career in mind. The author has studied students’ preferences of the final year project required to complete their degree at the University of Salamanca (Salamanca, Spain). It has been found that most female students are choosing a more theoretical final project instead of a practical one relevant to professional employment, contrary to their male peers. Focus group meetings with the students showed that at the end of five years of engineering geology training, many female students, unsatisfied with the content of their courses, feel that their expectations had not been met. They often have preferences for traditional geology rather than applied branches of the subject. Also, they do not feel comfortable with future job prospects in the profession. From the findings of this research it is clear that tutoring and mentoring would be valuable from the beginning of studies to allow all students to become aware of the content and the potential outcomes of engineering geology studies. In the case of female students, it is particularly important for them to know from the very start that they are about to join what is still a man’s world but that they are capable of achieving just as much as men can in the profession. Most importantly, the involvement of more female engineers in professional engineering, including teaching duties, should serve as example and role models in students’ education and future careers.

## 1. Introduction

**Figure 1:**
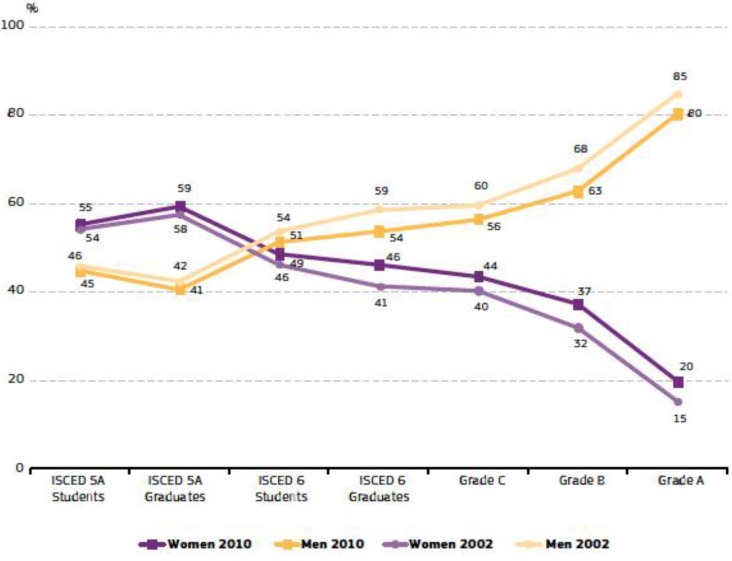
Proportions of men and women in a typical academic degree course, students and academic staff, EU-27, 2002–2010. ISCED: International Standard Classification for Education. In the case of Spain, for academic staff: A = Full Professor; B = Tenured Lecturer; C = Assistant Lecturer; ISCED 6 = PhD student. Complete information in [2].

Although the general gap for women in scientific studies and professional careers has gradually changed in recent years, the balance of their contributions is still negative [1,2,3,4]. There is indeed strong vertical segregation in the academic world, but the situation varies considerably, depending on the field of science considered. The most imbalanced situation, with very little change over time, is found in engineering studies [1,2,5]. Women have gained ground in education to an impressive extent, but progress has been uneven in science in general, and in engineering in particular. In North America, geological engineering has the lowest percentage of participation of women in geoscience occupations [3]. European commissions have investigated this issue and the conclusion is that current women’s representation in companies and universities within the science and technology sectors is still unacceptable [2,6]. In these reports the “leaky pipeline”—that is the scissors diagram that shows the drop-off in the number of women in science as they move higher up in the academic career, despite their decent achievements and better results than men during the early career stage—is clearly visible on comparing the proportions of men and women in a typical academic degree course. This plot shows the evolution of students and academic staff in the 1999–2003 period. The “She Figures” Report for 2012 shows the same scissor picture for the 2002–2010 period (Figure 1) [2]. The figure shows that female students are present in higher proportions than men during their graduate studies. It is possible to note the positive evolution from 2002 to 2010. However, the scissors cross once the students reach the stage of preparing their doctorates and persists during the ensuing levels that open the way to academic and research careers, leaving a very low proportion of women (around 20%) at the very top. The same has been reported in USA geoscience academia [7,8]. In Spain, the percentage of full professorships occupied by women in all areas is 19.7% [1].

**Figure 2:**
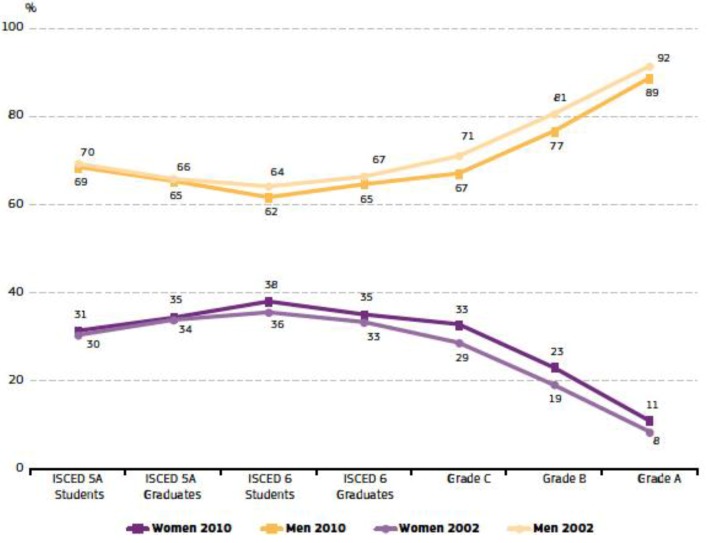
Proportions of men and women in a typical academic degree in science and engineering, students and academic staff, EU-27, 2002–2010. Code explanation as in Figure 1.

However, what is really striking is the figure for engineers. For this group, the scissor lines do not cross at all, and female students and female academics always constitute a minority. The destruction of women’s positions becomes increasing pronounced at each stage above the PhD level and the evolution with time is too small and too low to be considered positive (Figure 2). Thus, the result from this official study is comparable to that observed in the present work as regards the lack of female engineers who can act as role models for female students, allowing the picture in professional engineering geology outcomes to change (see below).

Statistically, what the “She Figures” official report finds is that women in science and engineering account for only 31% of the student population at the first level. This percentage increases with level, rising to 38% of women at the student PhD level and 35% at the graduate PhD level. The report finds that the lack of appeal for young women of science and engineering studies is particularly problematic during the earliest stage of a typical academic degree in this field, since women tend to be better represented among PhD students and graduates. However, the same is not the case for engineering geology at the University of Salamanca (USAL). In this case, it is during the last stage when female students desert the professional engineering aspects of their studies. In fact, the Eurostat Commission Report for early leavers from education and training for the year 2013 reflects an increase in Spanish women (19.9%) with respect to the target for the country (15.0% for women) and the total for the whole EU-28 (11.9%, including men and women). The good news is that this proportion has shrunk considerably in recent years, although segregation by gender is only considered in this 2013 final report [9].

The same pattern for academic degrees in science and engineering as in all fields of study was noted when going higher in the status scale. Of 35% of female PhD graduates, the proportion of women drops to 33% in grade C academic staff, 23% in grade B and just 11% in grade A. This trend has been noted by studies across Europe and in the USA [4,8]. Women’s participation in science and engineering is thus, comparable to the situation in all study fields taken together. Comparison of 2002 and 2010 points to an improvement in the proportion of female scientists and engineers that is slightly less pronounced than for all study fields taken together. For the present study, it should be highlighted that none of the female instructors in engineering geology in Salamanca occupies the highest position: full professorship. In fact, there are no full professorships occupied by women at the geology department of USAL.

Geology has been a degree at USAL since the 1970s. The geology degree teaches students about the geological processes that govern the evolution of the Earth’s surface and interior. It covers the essential Earth science disciplines, such as the sedimentary, igneous and metamorphic origins of rocks and minerals; the evolution of life on Earth; plate tectonics and the structural deformation of rocks; fieldwork; geological mapping; and natural hazards. In 2000 the study of engineering geology was established at this university, sparked by a strong demand for these studies due to the prosperous construction industry established in Spain during those years, with the ensuing disastrous consequences related to the now well-known economic crisis that has affected the building sector. Engineering geology is the application of geological principles to civil and mining engineering (amongst others). Architecture and engineering studies in Spain were the only degrees that demanded a final year project to complete the degree course [10,11]. However, the European Higher Education Area (EHEA) introduced some changes in student curricula for the Bologna-adapted system, and one of these changes is related to the completion of a final project for all university degrees [11]. This final project can be defined as an assignment to be completed by students during the last academic year of their degree courses (*i.e.*, in the fourth year of the Bologna-adapted system), and its purpose is to assess students’ general competencies in the subjects of the degree course [11]. The final project must carry between 6 and 30 ECTS credits (ECTS refers to the European Credit Transfer System, the standard system for comparing the academic achievement and performance of students in higher education across the European Union). In Salamanca, engineering geology has recently been adapted to the new Bologna system and it is now time to reflect on gender issues (e.g., unbalanced gender composition of teachers in departments, selection of more technical final projects by male students compared to female students) observed during the years of the implementation of final projects in the non-adapted engineering geology degree in Salamanca to extrapolate the results from other schools, and to compare the situation in Spain to what is happening in other countries.

The present work focuses on the “leakage” of female students towards less technical topics at the end of engineering studies. More precisely, the research questions prompting this study are as follows:

1. Why do female students enter a bachelor’s degree in engineering geology if they “leak out” at the end of their studies?
2. What can be done to encourage more female students to take engineering degrees in general and engineering geology in particular, with a professional engineering objective in view?

The study was done using USAL as an example, where geology and engineering geology are currently offered as four-year degrees, although before the Bologna system was applied (when this research was conducted) they were five-year degrees. At that time (with five-year degrees), only engineering geology demanded a final project to obtain the final degree certificate. The thematic final projects chosen by female students prompted the author to undertake this study.

The author anticipates that the conclusions drawn from this pilot investigation will unveil that our female students tend to opt for less professionally oriented technical projects that will eventually offer them fewer possibilities in technical professions. This study may also contribute to spread the reality of the minority of women in engineering programs in general, and engineering geology in particular, and to proposing possible solutions to change the composition of the professional engineering context in terms of gender that can be considered as reflecting a geoethical issue.

## 2. Methodology

### 2.1. Study Design and Sample

Engineering geology studies at USAL started in 2000. The degree used to have a six-credit final work project within the previous non-adapted curriculum. In the new, adapted system, which started in Salamanca in 2010, a minimum of 12-ECTS credits project is now compulsory for all Spanish university degrees. In non-adapted degrees, engineering geology studies had a duration of five years, but this has been reduced to four years with the new Bologna system [11].

A total of 97 students completed their final projects to gain their engineering geology degree during the studied period. Out of that total, 44 were female students and 53 male students. This means that over 40% of students at this level were female, which is more than the 31% observed in the European “She Figures” report [2] addressing engineering studies. The engineering geology teaching staff at USAL (during the study period) comprised 51 instructors (33 men/18 women) (Table 1).

**Table 1:** Background formation concerning the teaching staff in engineering geology studies at the university of Salamanca.

Teaching Staff Background	Male	Female
Geology	17	11
Engineering	10	1
Mathematics	1	2
Physics	4	2
Chemistry	0	2
Economics	1	0

Forty-one members of the staff had tenure (only five male and three female instructors had part-time positions and at the moment of writing up this study two of them, one male and one female, were no longer working in the geology department). There were 36% of women in the group of tenured professors, which is much higher than the 23% described in the “She Figures” report for science and engineering degrees. However, within this teaching group only one female teacher belonged to an engineering department (Table 1). In the same teaching group, 10 male professors (22%) are from an engineering department. In total, 28 members are part of the geology department (over 50% of the engineering geology teaching staff).

Because of the lack of experience in engineering geology studies at USAL and the scarcity of engineering professionals within the teaching staff, final projects have often been misunderstood by the teachers as true research projects (the equivalent to minor theses in university degrees). These may take students a fairly long time to complete, demanding extra effort from them relative to the credits earned [11]. The responsibility of professors and tutors in these studies is to supervise student projects that can be concluded easily within the time established to earn the project credits (1 pre-ECTS credit = 10 h in non-adapted studies; 1 ECTS credit = 25 h in adapted studies). The achievement of a timely end of these projects may require a change of approach in both students and the teaching staff. It is crucial for academic administrators to inspire and pick up on this approach change for the new adapted degrees. For students to start their final project and for their registration as candidates for projects they are required to have successfully passed all the other subjects of the degree course. The project may be linked to any of the different areas of knowledge that have been taught during the degree. Spanish university regulations state that a final project can be directed towards either a professional (technical) or more research-oriented work (*i.e.*, on mineralogy, palaeontology, petrology, stratigraphy, volcanology and so on in the case of engineering geology). During the project, students must demonstrate their knowledge, skills and abilities in one or more subjects taken during their degree studies. The subjects for final projects can be offered by the teaching staff or may be chosen directly by students under the supervision of their instructor.

### 2.2. Study Period and Data

The study was conducted at the school of sciences at USAL over seven years (from 2007 to 2014) using engineering geology data relating to the final projects prepared and defended. For the present work, 97 final projects, which were the total defended projects between the 2006–2007 to 2013–2014 academic years, are analysed in terms of student gender, tutor gender and the thematic subjects of the projects. These data were collected from the administration office of the school of sciences of USAL, where students have to file the application forms for the defence of their final projects. Descriptive statistics were applied to the results to be able to compare with other similar studies conducted in Europe and North America.

Focus group meetings and individual interviews were organized with male and female students to discover their reasons for choosing engineering geology studies instead of geology. The focus group included ten female and three male students who had chosen to prepare their final project under the author’s supervision. The focus group meetings took place during 30 to 45 min in a seminar room to give an official character to the event but not too formal as a tutorial session, which normally takes place at the supervisor’s office, to make the students more comfortable and the subject easy to discuss. Basically, the author listened to the students, taking notes on their reactions to some of the open-ended questions such as: what factors encouraged you to select engineering geology as your major? Or what is your perception of women’s status in engineering geology? The individual interviews produced important data regarding the effect of the teaching staff on the students. Some of the female students admitted that they felt intimidated by some of the professors when they were not able to follow some of the more technical parts of the subjects.

To complete the dataset for conclusions, the author has added data from observations made during a European summer school (an ERASMUS Intensive Programme) coordinated by herself. While doing the research on the behavior of male and female students at the end of their engineering geology studies for this paper, the author observed that the ERASMUS Intensive Programme student enrolment was following the same path regarding the biased selection of less technical courses by these European female engineering students.

To guarantee trustworthiness of the qualitative analysis the author has made sure that the four criteria proposed by [12] are accomplished: credibility, transferability, dependability and confirmability (in preference to objectivity).

## 3. Results and Discussion

As mentioned in the Introduction, tutoring final projects was voluntary during the non-adapted studies. Tutors were responsible for vetting an appropriate subject and they guided students through to the completion of the final project. This task often involves laboratory and fieldwork. Of the 51 people teaching in engineering geology, 27 (around 50%) had tutored final projects during the study period, but only seven of them were women (12% of the teaching staff in engineering geology). Thus, 20 men (≈26%) had tutored final projects, and most engineering-based projects were tutored by men (in fact, all except one, which was co-tutored by the only female engineering professor and two other male engineering colleagues). This observation will be important when discussing role models in the sections below.

Table 2 shows that, of the total number of projects defended in engineering geology, 67 were engineering-based (*i.e*., around 70%). However, fewer than 30% of these projects were presented by female students. This means that most male students of the study group chose to complete their degree course with a project focused on technical engineering, while their female peers took a more “scientific” subject.

**Table 2:** Themes chosen by female and male students for their final projects.

Final Project Subject	Male	Female
Geology	6 *	24 **
Engineering	47	20

Notes: * 3 of them were tutored by the author. Subject chosen by the student after a tutoring session; ** 10 of them tutored by the author. Subject chosen by student after a tutoring session.

The first option is logical because there also is a geology degree that the students could have chosen if that was indeed their professional wish. However, in the present study it was evident that fewer than half of the female students ended up with an engineering-oriented final project, while the majority opted for a geology-based one—*i.e.*, a less technical project—to complete their training.

**Figure 3:**
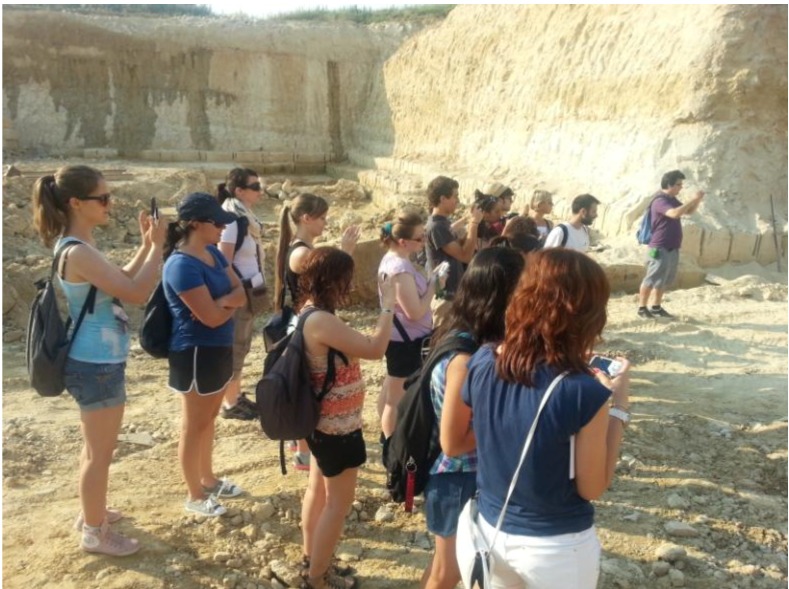
Students of the ERASMUS IP 2013 visiting a sandstone quarry.

To illustrate the gender-imbalanced involvement of female engineering students in geology-based subjects, the author of this paper wishes to add the observations made during an ERASMUS Intensive Programme (IP) on architectonic, cultural and historical heritage, coordinated by the author, which took place in the summer of three consecutive years, namely 2011, 2012 and 2013 [11]. This IP was intended for final-year students, master’s students and PhD students from the universities of Salamanca, Coimbra, Ferrara and Budapest who were interested in the offers of the IP. Since all the European co-coordinators of the IP were geologists who taught in engineering degrees in the four European countries listed above, most students were related to these degrees, all of them technical, except for one male student (Brazilian in origin, but a student at USAL) from fine arts who joined the first call. Table 3 shows the composition of the groups. It can be seen that most of the participants were women (Figure 3); although attempts were made to make the gender composition of the groups equal, very few male students applied to participate. Coimbra was the university that had the most balanced composition in such participation. So far, we do not have an explanation for this, but it is worth noting that many of the female participants from engineering geology in Salamanca ended up doing a final project tutored by the coordinator of the IP and author of this study (e.g., [13,14,15,16,17,18]).

**Table 3:** Gender composition of the ERASMUS Intensive Programs celebrated during the summers of 2011, 2012 and 2013.

Origin of students	Male	Female
Salamanca	5	10
Coimbra	7	6
Ferrara	5	10
Budapest	5	16
Total	22	42

### 3.1. Explanations for the Decision of Female Students When Choosing a Final Project Subject

First, an analysis must be performed of the reason why during the first stages of engineering geology almost the same number of male and female students can be found, whereas the number of female students in the final years is reduced, and many decide to end up the engineering geology studies with a less technical final project. An explanation could stem from a failure to choose the “correct” degree course, and in this decision family influence should be considered. According to some of the female students during the tutorial sessions and focus group meetings, they enrolled in engineering geology studies with the belief that being an engineer is recognized as “having more worth” than having a scientific, non-engineering degree (e.g., geology) [19]. This idea is often triggered by the opinion of the student’s family, since it has been shown that family opinion is always very important for students in deciding which degree to follow [5,13]. Having the two degrees at the USAL led the author to reflect on why female students did not take geology instead of engineering geology if they were going to end up with a non-technical project that would not place them in the path of what they would be doing if they became engineers. In the female students’ own words during the focus meetings, in the first years of their degree courses they realize that they are immersed in a man’s world; they are not taken seriously in debates and discussions, and are even frowned upon by some male teachers because they do not achieve the expected results in some of the most technical subjects. Some authors have described how female students leave engineering studies in response to mismatched expectations [19,20,21]. Our female students tend to finish their studies, but many definitely are not interested in their potential as future engineers, as demonstrated by choosing less technical final projects.

Another explanation for the attitude of female students when making the decision to embark upon a geology research-based project is based on the lack of role models in engineering geology [22,23]. The marked minority of female engineers in the teaching staff could be interpreted by female students as a specular image of the professional future they envisage for themselves, which could derive from an ethical issue related to the lower academic status of female teachers who could serve as role models. Negative perceptions of female academics in senior positions may in some cases also serve as disincentives against pursuing an engineering academic career, since female students are discouraged by the perceived lifestyle of senior female academics in their chosen field [24,25]. Young female students look for a role model in all degrees. Female engineers as leaders of a team would be the engineers that many female students would wish to emulate. The existence of this leadership is probably one of the explanations for the high enrolment of women in degrees that offer student mentoring by women, such as degrees in the life sciences-medicine, dentistry and pharmacy- and several other areas in the sciences that break the rules of women’s involvement in scientific careers [1,2]. It has been shown that female graduate students look forward to having mentors during their degree studies, but particularly female mentors, who would reflect the great professional diversity in women’s lives today [15,23]. The lack of female role models continues to hinder advances for women, so they find themselves “establishing their own way and styles that work for them, and as a result, the additional reinforcement from a mentor is useful [26].

### 3.2. Proposed Solutions Based on Experiences

The observations at USAL are comparable to those reported throughout Europe and North America [1,2,3]. Positive actions have been taken at many institutions to promote the involvement of women in engineering studies across the world. Although it is still early to analyse whether the implementation of these positive actions has afforded better results in making engineering studies more appealing to female students, below are some actions proposed for the engineering geology Spanish study group, based on what has been done elsewhere for comparison in future studies.

#### 3.2.1. Action 1: Promoting Engineering Programs Sponsored by Private Companies

Some universities in North America are obtaining infrastructure and economic help from companies in the private sector to promote engineering programs among women. Such is the case of Edison, one of the nation’s largest electric companies, and California Polytechnic Pomona [27]. Through this collaboration they organize networks, chats and other ways of interaction among female engineering students, female mentors, faculty members and industry workers to share their knowledge and experience with the students. It has been demonstrated that women think in different ways when organizing a project, sometimes paying more attention to details that men may not take into account. Companies can recognize the skills needed for future workers, including female engineers. In fact, specific research along this line is being promoted in the Horizon 2020 program [28].

#### 3.2.2. Action 2: Holding a Girls’ Day

Society is still full of traditional sex-role stereotypes [29] and this is reflected in the enrolment in some degrees in science and engineering [30,31]. To avoid this tendency, many American and European universities organize an event related to engineering studies: Girl’s Day. This movement started in the 1990s and shows young women how creative and collaborative engineering can be and how engineers are changing our world. This activity was imported into Europe [32] (first in Germany and Austria) in the early part of the present century, and the idea is to promote a reversal of trend in the choices of degrees by young women and to alter their perspectives regarding employment. It is very important to give young women appropriate information and support when it comes to them making their choice from across the entire degree spectrum and selecting a job that corresponds to their interests and talents and one that they would enjoy. Here, should be added the importance of role models for young female students. Some Spanish universities have been celebrating a Girls’ Day for the last five years (e.g., Barcelona, Oviedo, the Canary Islands and Zaragoza [33]). The objective sought by these universities is to awaken young women’s interest in engineering degrees and jobs to draw the attention of companies to the potential of young female scientists, and to sensitize parents and the public at large to the fact that the demand for young women in traditionally male-dominated jobs is increasing. Thus, it is not a theoretical event, but a practical one in which young women and men are given the opportunity to become actively involved in the tasks of a typical working day in an engineering company or laboratory, to experiment freely, and to strengthen their self-confidence and trust in their own skills. La Laguna University in the Canary Islands offered a Girls’ Day project during the 2013–2014 academic year and the present one, 2014–2015, supported economically by the Spanish Ministry of Health, Social Services and Equal Rights, the National Women’s Institute and the local government of the Canary Islands, and endorsed by the Spanish Association for Women in Science and Technology (AMIT) [34]. During the events, secondary school students attended seminars to break role and gender stereotypes in the choice of studies and degrees and to attract female students to certain degrees and branches offered at the University of La Laguna, especially technical studies. The students also benefited from a visit to the Canary Islands Astrophysics Laboratory and to the Technological and Scientific Campus of Tenerife, where female scientists explained their own research work. These actions would help to stimulate young women and to change the position of the lack of appeal in science and engineering studies at the earliest stage. Girls’ Day fulfils the ambition to actively recruit female students in the major, as [5] and [35] recommended, in order for science departments to become female-friendly.

#### 3.2.3. Action 3: Implement Equal Rights Legislation at the University

Most countries around the world have gender equality laws. Spain has had its own legislation since March 2007 [36]. The purpose of these laws regarding the issue under consideration here is to promote gender equality, including equal integration and equal opportunities in all functions within public institutions, such as universities. This should be reflected in the composition of committees and top-level academic staff positions. However, in many of our institutions this is not happening. The public authorities have the duty to promote gender equality and incorporate gender equilibrium in all planning and administration tasks, taking measures to remedy the situation where necessary. So far, however, they have only shown a paternalistic attitude towards the complaints presented by university members who note that the 2007 Equal Rights Act is not being implemented, and commissions and boards are often blatantly selected with a gender imbalance. Although studies have shown that affirmative actions, based on merit, is better at rewarding and promoting talent than the more passive policy of equal opportunities [36], if equality commissions at universities were obliged to present a reflected report with the real figures of gender equality at the different levels, one would see the glaring underrepresentation of women at high levels in the academic world, mainly in technical studies, that is responsible for the lack of female role models for students. Changing these figures would certainly help to change our female students’ ideas about engineering as a professional livelihood.

#### 3.2.4. Action 4: Promoting the Involvement of Female Teachers in Tutoring Final Projects

In this case study, it was observed that over 26% of male teachers from the whole teaching group were involved in tutoring final engineering geology projects, while only 12% of female teachers had tutored final projects. It should be noted that in many contexts the involvement of women in changing the equality picture is vital, but the tutoring of final projects has been on a voluntary basis up to now. In order to analyse the role of women in the decisions of female students regarding their professional future, they should become actively engaged in all the different stages of women’s education. Engineering geology degree coordinators should work on the imbalanced situation observed at the tutoring stage, further exacerbated by the very low proportion of female teachers in this study area at USAL. The ratio of female staff with tenure in engineering and architecture in Spain is 39.7%. In engineering geology in Salamanca, 35% of the teaching staff are women, most of them with tenure. However, only one of them has an engineering background. Again, it is recalled that none of these female professors holds the highest academic position. Taking into account that the final projects are either offered by teaching staff or requested by the students themselves to a particular member of the teaching staff, and that only 12% of the total of the female teaching staff was actively involved in tutoring, the author suggests that the promotion of involvement of women in tutoring and monitoring the degree course work of female students would trigger the development of a true female engineering network, helping both future female engineers and the implementation of female role models in engineering geology. However for this, more female engineers must be contracted for teaching engineering geology. The existence of social and professional activities led by a female faculty member as part of her departmental service, not as a voluntary activity, would help female students feel confident about their career future [35]. Moreover, research has found that small improvements in the culture of a department can have a positive effect on the recruitment and the retention of female students, and that departments that work to integrate female faculty and enhance a sense of community are also more likely to recruit and retain female faculty [5]. As [37] pointed out, educators at all levels of science, technology, engineering and mathematics (STEM) disciplines should be aware that a wide assortment of behaviors they may unknowingly display create a chilly classroom or unacceptable academic environment for their female students.

## 4. Conclusions

The author of this paper triggered a discussion through the research questions related to why female students enter a bachelor’s degree in engineering geology if they “leak out” at the end of their studies, and to solutions to encourage more female students to take engineering degrees in general and engineering geology in particular, with a professional engineering objective in view. Through that discussion, it has been shown that female students do not feel comfortable with the present teaching conditions, including the lack of role models to follow, and that they enter engineering geology studies following a wrong idea, supported sometimes by their own family’s advice.

There is strong statistical evidence that the modern university has discriminated against women in tenure and promotion decisions [1,2,3]. It also is clear that efforts to recruit women into technical programs and to eliminate obstacles within the education system, while obviously necessary, are insufficient. Employment and promotion policies must be revised to ensure that qualified women have a reasonable chance of pursuing a rewarding career in engineering. Affirmative action, based on merit, has been shown to be a suitable way of drawing close to equality in both employment and education, since it has been demonstrated that organizations need a variety of different talents that are represented by both women and men [38,39]. This will change the ideas of our female students in engineering degrees in general, and engineering geology in particular, such as the study case at USAL.

In high school, although half of the teachers are women, very few of them teach the more technical subjects (e.g., mathematics, physics, *etc.*). It is here where the problem begins, where female students lack female role models to follow; they seem to be unable to find self-esteem as professional scientists. When asking these students about female leaders in science, they almost unanimously cite only the figure of Marie Curie. Not even her daughter, Irene Jolliot-Curie, is mentioned. The lack of information on women’s achievements in science can affect girls’ performance and their aspirations [5]. Several studies and reports [40,41] have shown that female students who are apparently well qualified and strongly motivated during college and graduate school, lose their self-esteem, are harassed by male professors and are excluded from crucial discussions. This makes them feel that they do not belong [42]; in [43] the author explained that self-confidence has an influential effect on a woman’s choice of a career in the sciences. In [44], the authors performed a periodic survey of how female students feel about working in a specific environment and the results could be informative as regards what these students believe they are contributing to their specific engineering degree and profession. Although these references are a few years old, the conclusions are certainly not out-of-date. There are still cases in scientific degrees in which women feel out of place [35]. Nokes and Gustafson [44] found that recruiting women into engineering programs is sometimes not the problem: it is keeping them there until the end of the degree that has been more challenging. This is the same outcome as observed in engineering geology at USAL. The perspective approached by the Young Earth Scientists (YES) network, linking early-career geoscientists through scientific research and interdisciplinary networking, would be a further step in analyzing the circumstances in different countries. YES aims at providing professional development resources to prepare young scientists for integration into the workforce and to prepare them to serve as key leaders and advisors in the area [45]. A gender component in this approach would provide new perspectives in geoethics to offer female students the possibility of finding role models who can be followed, entrusting their technical professional careers to them.

The author wishes to make a case for affirmative action to reinforce the role of women in teaching science in general, and in engineering degrees in particular, in order to meet equal rights legislation when setting up commissions and management boards. These actions must aim at an increase in the ratio of female teachers in these studies; an increase in their participation in tutoring young female students; and to create female role models to help female students to feel more confortable in a still male-dominated world. Besides, positive actions, such as the implementation of activities like the Girls’ Day, with collaboration from high schools, universities and companies that have female professionals in their boards of management will encourage more female students to enroll in these technical degrees. This will allow women working in science to share their concerns and will underscore the importance of gender issues in the real world. The relatively recent history of engineering geology studies at USAL furnished a relatively small number of cases for the study, therefore limiting the study’s conclusions. It would be worthwhile to perform a follow-up of the issue in forthcoming years.
